# Maternally Instigated Diapause in *Aedes albopictus*: Coordinating Experience and Internal State for Survival in Variable Environments

**DOI:** 10.3389/fnbeh.2022.778264

**Published:** 2022-04-25

**Authors:** In Hae Lee, Laura B. Duvall

**Affiliations:** Department of Biological Sciences, Columbia University in the City of New York, New York, NY, United States

**Keywords:** diapause, *Ae. albopictus*, reproduction, mosquito, seasonal change

## Abstract

The Asian tiger mosquito, *Aedes albopictus*, is one of the most dangerous invasive species in the world. Females bite mammalian hosts, including humans, to obtain blood for egg development. The ancestral range of *Ae. albopictus* likely spanned from India to Japan and this species has since invaded a substantial portion of the globe. *Ae. albopictus* can be broadly categorized into temperate and tropical populations. One key to their ability to invade diverse ecological spaces is the capacity of females to detect seasonal changes and produce stress-resistant eggs that survive harsh winters. Females living in temperate regions respond to cues that predict the onset of unfavorable environmental conditions by producing eggs that enter maternally instigated embryonic diapause, a developmentally arrested state, which allows species survival by protecting the embryos until favorable conditions return. To appropriately produce diapause eggs, the female must integrate environmental cues and internal physiological state (blood feeding and reproductive status) to allocate nutrients and regulate reproduction. There is variation in reproductive responses to environmental cues between interfertile tropical and temperate populations depending on whether females are actively producing diapause vs. non-diapause eggs and whether they originate from populations that are capable of diapause. Although diapause-inducing environmental cues and diapause eggs have been extensively characterized, little is known about how the female detects gradual environmental changes and coordinates her reproductive status with seasonal dynamics to lay diapause eggs in order to maximize offspring survival. Previous studies suggest that the circadian system is involved in detecting daylength as a critical cue. However, it is unknown which clock network components are important, how these connect to reproductive physiology, and how they may differ between behavioral states or across populations with variable diapause competence. In this review, we showcase *Ae. albopictus* as an emerging species for neurogenetics to study how the nervous system combines environmental conditions and internal state to optimize reproductive behavior. We review environmental cues for diapause induction, downstream pathways that control female metabolic changes and reproductive capacity, as well as diapause heterogeneity between populations with different evolutionary histories. We highlight genetic tools that can be implemented in *Ae. albopictus* to identify signaling molecules and cellular circuits that control diapause. The tools and discoveries made in this species could translate to a broader understanding of how environmental cues are interpreted to alter reproductive physiology in other species and how populations with similar genetic and circuit organizations diversify behavioral patterns. These approaches may yield new targets to interfere with mosquito reproductive capacity, which could be exploited to reduce mosquito populations and the burden of the pathogens they transmit.

## Introduction

**FIGURE 1:**
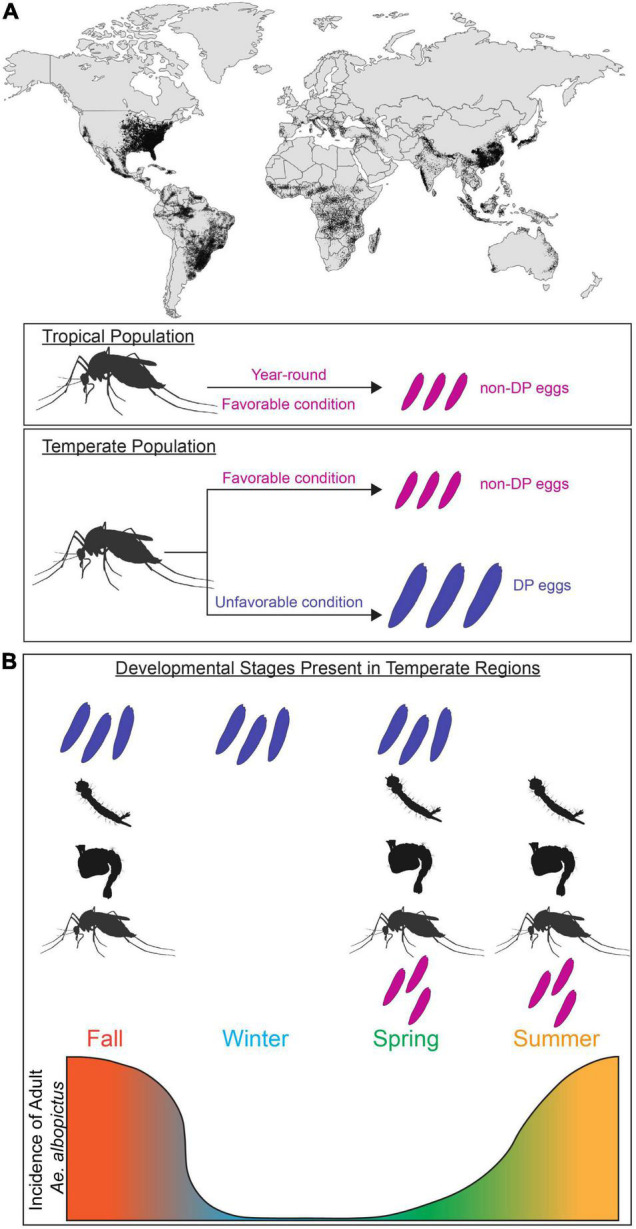
*Aedes albopictus* global invasion and population expansion in temperate and tropical regions. **(A)** Global map of predicted distribution of *Ae. albopictus* population (adapted from Kraemer et al., 2015). Adult females found in tropical regions lay non-diapause eggs (magenta) majority of year-round. In temperate regions, adult females found in favorable conditions (magenta) lay non-diapause eggs. In unfavorable conditions, adult females alter their reproduction (blue) and lay diapause eggs (blue). **(B)** Incidence of *Ae. albopictus* at different life stages in temperate regions. Temperate population have unique subsets of developmental stages present (diapause and non-diapause egg, larva, pupa, adult) in each season. Incidence of adult *Ae. albopictus* in temperate region is depicted by the gradient graph.

Insects are successful across huge geographic ranges due to their capacity to physiologically and behaviorally adapt to a wide variety of environments. In tropical climates, the annual range of temperature is usually small; however in temperate climates the environmental conditions optimal for survival prevail during specific seasons. This requires animals to energetically provision themselves or their offspring to successfully endure harsh winters (Tauber and Tauber, 1976). To determine the appropriate reproductive behavior and allocation of nutritional resources, this adaptive response requires the nervous system to integrate environmental cues and internal state (gravid or not). Understanding how animals adapt to survive unfavorable environmental conditions is critical for understanding their behavior, distribution, and population growth as well as speciation and interspecific interactions. This is particularly important for *Ae. albopictus* (Skuse, 1894), a highly invasive mosquito vector of arthropod-borne disease that has expanded its range to include temperate and tropical regions on every continent except for Antarctica (Bonizzoni et al., 2013; Figure 1A). Climate change modeling predicts that these mosquitoes will continue to expand and redistribute their geographical range, increasing net and new exposure to *Aedes*-borne pathogens (Ryan et al., 2019).

Entering the state of preprogrammed developmental arrest called diapause is a successful strategy to survive harsh environmental conditions. Diapause is a dynamic process that includes several phases; pre-diapause stages include “induction” and “preparation” and diapause itself can be divided into “initiation,” “maintenance,” and “termination” stages (Koštál, 2006). During diapause induction, which occurs well in advance of seasonal change, animals are sensitive to cues that predict the arrival of unfavorable environmental stressors. Generally, this only occurs at a genetically predetermined life stage. This sensitive stage may take place within the lifetime of the diapausing individual or, as in the case of *Ae. albopictus*, in the preceding generation. Seasonal shifts are likely anticipated by measuring daylength (photoperiod). Both the shortening of daylength and static short photoperiod induce diapause, causing individuals to undergo physiological and behavioral changes. Next is the preparation phase, when individuals provision themselves or their offspring by accumulating lipids, proteins, and carbohydrates. Once diapause is initiated, development is arrested and diapause is maintained as animals exhibit reduced metabolism, developmental arrest, and increased sensitivity to certain environmental cues (such as light and temperature) in preparation for diapause termination phase. Diapause termination occurs spontaneously in some species, but in others, the resumption of development may be initiated by external stimuli, which signal the eventual return of favorable conditions (Golden and Riddle, 1984; Sommerville and Davey, 2002; Milonas and Savopoulou-Soultani, 2004). This period of dormancy is characterized by changes in insulin signaling, metabolism, cell-cycle arrest, and stress-response genes (Lees, 1955; Hansen et al., 2011; Poelchau et al., 2013a). Diapause has independently evolved multiple times and can occur at distinct developmental stages (embryonic, larval, adult). Typically, a species is only capable of entering diapause at a specific developmental stage, one factor that differentiates diapause from quiescence, which is a dormant state that can occur during any developmental stage and can be quickly terminated upon exposure to favorable conditions (Tauber and Tauber, 1976).

Mosquitoes employ a diverse set of diapause strategies; *Wyeomyia smithii* enter diapause as larvae living inside pitcher plants (Bradshaw and Lounibos, 1977), whereas *Culex* mosquitoes enter diapause as adult females (Eldridge, 1987). In this review, we focus on *Ae. albopictus*, which exhibits maternally instigated embryonic diapause. In temperate populations of *Ae. albopictus* where fall/winter conditions are too harsh for adult survival and reproduction, pupae and adult females detect seasonal changes in daylength and produce diapause eggs that enter developmental arrest as pharate larvae inside the chorion of the egg (Wang, 1966; Mori et al., 1981; Denlinger and Armbruster, 2014). These developmentally arrested pharate larvae remain non-responsive to hatching stimuli for a period of time, thus preventing autumnal hatching and larval mortality during cold winter conditions. Under long day photoperiods similar to those found in spring and summer (e.g., 16 h light:8 h dark), females from temperate populations produce non-diapause eggs that complete embryonic development and are immediately responsive to hatching stimuli (Figure 1A). In tropical environments, temperatures are permissive for reproduction year-round and tropical *Ae. albopictus* populations do not undergo diapause (Hawley, 1988; Pumpuni, 1989; Figure 1A). This results in seasonal differences in the relative abundance of immature and adults mosquitoes in temperate regions (Figure 1B). While ancestrally temperate and tropical strains can interbreed and share genetic and anatomical organization (Boyle et al., 2021), they differ in their reproductive responses to environmental cues. It is unclear if the physiological differences between ancestrally temperate vs. tropical strains or between females actively producing diapause vs. non-diapause eggs arise in neural or reproductive circuits, or elsewhere entirely. The evolutionary history of *Ae. albopictus* has resulted in populations with distinct responses to environmental cues, which provides researchers the opportunity to study neural circuits that control these responses in groups with behavioral variation.

## Evolutionary History of *Aedes albopictus* and Behavioral Variation in Diapause

Understanding the evolutionary history of *Ae. albopictus* is critical to inform our understanding of how variation in reproductive behaviors arose and has been maintained. In its ancestral range in Asia, *Ae. albopictus* is found in both temperate and tropical environments, spanning from India to Japan (Hawley, 1988), and this invasive species has now become established on every continent except for Antarctica (Lounibos, 2002; Bonizzoni et al., 2013; Figure 1A).

The first case of *Ae. albopictus* in the continental United States was reported in 1985, when an established population was discovered in Texas (Sprenger and Wuithiranyagool, 1986). Trade records implicate the use of imported water-containing tires as breeding sites in facilitating the dispersal of *Ae. albopictus* from a Japanese population (Sprenger and Wuithiranyagool, 1986; Hawley et al., 1987). These strains collected in Texas were sensitive to photoperiod and females exposed to short daylengths produce diapause eggs, suggesting that they originate from an ancestrally temperate population (Lounibos et al., 2003). Likely through multiple introductions and geographic spread, *Ae. albopictus* now inhabits temperate regions of the North American east coast and midwest, and subtropical and tropical regions in the Caribbean, Mexico and Central America (Moore, 1999; Lounibos et al., 2003; Bonizzoni et al., 2013). The transition of North American populations from temperate to tropical regions resulted in the gradual loss of an environmental requirement to enter diapause under natural conditions, with an environmental breakpoint found in northern Florida (Lounibos et al., 2003). However, females collected from tropical regions in Florida have been reported to produce diapause eggs in laboratory settings, suggesting that diapause-induction pathways remain intact in these animals.

At almost exactly the same time that the species was detected in Texas, *Ae. albopictus* was also first detected in Brazil, near Rio de Janiero (Forattini, 1986). While the exact origin of this invasion is unknown, Brazilian populations are thought to have originated from an ancestrally tropical population due to the absence of diapause induction in this sample (Forattini, 1986; Hawley et al., 1987). Although *Ae. albopictus* is now broadly distributed across the Amazon basin, they are thought to have remained genetically isolated from North American populations (Birungi and Munstermann, 2002; Bonizzoni et al., 2013). It remains controversial whether diapause competence can emerge from a founding population of tropical origin that does not produce diapause eggs. Although Lounibos et al. (2003) reported that females from temperate regions of Brazil were able to produce diapause eggs, despite the likely tropical origins of their founders, earlier findings suggested that short photoperiods were never capable of inducing diapause if the founding population was of a tropical origin and did not originally undergo diapause (Craig, 1993; Hanson and Craig, 1994). Interestingly, populations from similar latitudes in the northern vs. southern hemispheres showed noticeable differences in their ability to produce diapause eggs when exposed to short day conditions in the laboratory. Animals collected from the northern hemisphere showed higher rates of diapause egg production in laboratory settings compared to those collected from similar latitudes in the southern hemisphere although both groups showed significant heterogeneity in their responses (Lounibos et al., 2003). These findings provide evidence for the emergence of diapause in populations from an assumed tropical origin (e.g., subtropical/temperate Brazilian populations), and ongoing loss of diapause in now-tropical populations from a temperate origin (e.g., Floridian and Caribbean populations). Thus, diapause competence is thought to be determined by rapid evolution induced by the selective pressures of the local environment as well as whether the origin of the founding population is tropical or temperate. However, it is difficult to exclude the possibility of multiple establishment events. Within the United States, there is evidence for rapid evolution in diapause incidence and seasonal timing in *Ae. albopictus* populations, supporting the conclusion that these adaptive phenotypes are critical for the high invasion potential of this species (Lounibos et al., 2003; Urbanski et al., 2012). Recent work mapping putative diapause-associated SNP clusters throughout the *Ae. albopictus* genome suggests that the evolution of diapause in *Ae. albopictus* is polygenic (Boyle et al., 2021).

The evolutionary history of *Ae. albopictus* has led to widely distributed global populations with varying reproductive responses to environmental cues. These changes likely have a genetic basis and although these populations share genomic and anatomical commonalities, the basis for variation in diapause competence remains an area for exploration.

## Maternal Responses to Environmental Cues: Diapause Induction

### Environmental Cues

Appropriate diapause entry requires accurate anticipation of seasonal changes. As winter approaches, the days become reliably shorter; laboratory studies and observations in the field have implicated photoperiod as the major stimulus for diapause initiation (Denlinger, 1986; Armbruster, 2016). Critical photoperiod (CPP) is the daylength that induces diapause entry in at least 50% of the population. Although generally insufficient to induce diapause alone, lower temperature can interact with photoperiod to increase diapause incidence at a given daylength (Pumpuni and Craig, 1992). Reduced larval nutrition has also been shown to increase the CPP and diapause incidence, meaning that animals that experience early nutritional stress enter diapause at higher rates earlier in the autumn, suggesting crosstalk between nutritional state and diapause entry (Pumpuni and Craig, 1992). Other environmental cues that signal seasonal change are also thought to play a role in diapause initiation and are reviewed in Tauber (1987). The CPP and the temporal dynamics of diapause entry have implicated circadian clock cells as seasonal sensors, as they are poised to detect light/dark cycles and influence physiology (Armbruster, 2016).

### Circadian Clock Involvement

**FIGURE 2:**
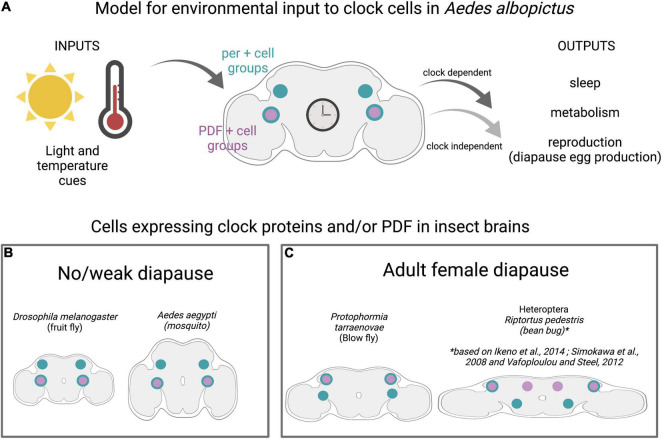
Clock cells in diapausing and non-diapausing insects. **(A)** Model for environmental inputs to predicted clock cell subgroups in *Ae albopictus* that are candidates for regulating diapause entry in adult females. Light and temperature cues are detected by central brain clock cells expressing the neuropeptide Pigment Dispersing Factor (PDF) shown in purple and/or core clock genes (denoted by period (per) in teal). Predicted clock subgroups are based on observations in *D. melanogaster* and *Ae. aegypti* (Baik et al., 2020). **(B)** Insects with no/weak diapause and those with adult female diapause **(C)** show conservation of clock anatomy, including distinct subgroups of central brain cells that express combination of per and PDF.

The circadian system has been suggested to play a key role in seasonal tracking, but the mechanism by which mosquitoes utilize this circuitry to interpret seasonal dynamics and initiate diapause entry remains unclear. There are three main models in the field. The first model is referred to as an external coincidence model, proposed by Bünning (1936). Bünning hypothesized that since the circadian clock already provides information related to light/dark cycles, organisms likely use their circadian clocks to measure daylength and initiate photoperiodic responses (Figure 2A). To detect long days, the light cue entrains the clock to determine a light-sensitive period in the late night and early morning. Diapause is initiated as the light-sensitive period shortens to match the critical photoperiod. In a contrasting internal coincidence model, proposed by Pittendrigh and Minis (1964), light entrains distinct dawn and dusk oscillators, so that a change in the phase of these two oscillators induces diapause entry. In the third model, called the hourglass timer or hourglass interval timer model, internal circadian clocks are not involved in initiating diapause and organisms measure day/night length using an independent system. For example, the accumulation of a chemical substance may initiate diapause after surpassing a critical threshold, or there may be an independent genetic basis for seasonal tracking (Bradshaw et al., 2003; Bradshaw and Holzapfel, 2010). These models may not be mutually exclusive but could occur in combination to mediate diapause initiation (Goto, 2013; Meuti and Denlinger, 2013).

Circadian rhythms are driven by endogenous molecular clocks, which consist of auto-regulatory loops of proteins that rhythmically repress expression of their own genes. Critical circadian genes include *period (per)*, *timeless (tim)*, *clock (clk)*, *cycle (cyc)*, and *cryptochrome (cry).* The genes and regulatory mechanisms of circadian rhythms are deeply conserved across the animal kingdom (Wager-Smith and Kay, 2000; Sandrelli et al., 2008; Beer and Helfrich-Förster, 2020). Neuropeptides, notably Pigment Dispersing Factor (PDF), coordinate circadian rhythms between clock cell subgroups in the insect brain. Despite this general conservation, there are some features in which *D. melanogaster*, the most well-characterized organism, is the outlier among insects. Specifically, *D. melanogaster* has a single cryptochrome (CRY), whereas most insects, including mosquitoes, have both light-sensitive and -insensitive cryptochromes (Gentile et al., 2009; Baik et al., 2020; Beer and Helfrich-Förster, 2020). Although the total number of clock cells varies widely between species, ranging from ∼80 to 90 in *Aedes aegypti* and *Anopheles coluzzi*, to 150 in *D. melanogaster*, and ∼400 in *Apis mellifera*, there is a similar pattern of clustering into anatomically distinct subgroups. Interestingly, PDF seems to play a conserved role in communicating across these subgroups and brain regions (Figure 2B; Vafopoulou and Steel, 2012; Fuchikawa et al., 2017; Beer et al., 2018; Baik et al., 2020) and PDF-expressing neurons have been implicated in mediating photoperiodism in multiple species, notably *Riptortus pedestris* and *Culex* mosquitoes. However, PDF may perform this role independent of its circadian functions (Ikeno et al., 2014).

Studies performed in multiple species indicate that functional circadian clock components are required for appropriate diapause entry (Denlinger and Armbruster, 2014) by detecting and regulating photoperiodic entrainment to appropriately sense daylength and signal reproductive system (Tauber, 1987; Armbruster, 2016). The circadian system has been linked to diapause regulation in multiple insects, and a recent study from the Arctic archipelago of Svalbard proposes that seasonal synchronization is dependent on the circadian clock in birds, even under constant light conditions (Appenroth et al., 2021). In *Drosophila triauraria*, allelic differences in SNPs and deletions in *tim* and *cry* between diapausing and non-diapausing strains are associated with diapause incidence although genetic linkage analysis suggests that *tim* and *cry* have independent effects on the occurrence of diapause, unlike their action in the circadian clock (Yamada and Yamamoto, 2011). In the bean bug, *R. pedestris*, *per* and *cyc* genes modulate diapause induction, and neurons in the pars lateralis are involved in photoperiod responses (Shimokawa et al., 2008; Ikeno et al., 2010). Studies in *Cx. Pipiens* have revealed the presence of an oscillating circadian network that is essential for diapause initiation in these mosquitoes (Meuti et al., 2015). The neuropeptide PDF is secreted by specific clock cells, regulates circadian- and light-mediated behaviors in *D. melanogaster*, and initiates diapause entry in the blow fly *Protophormia terraenovae* (Hamanaka et al., 2005; Shiga and Numata, 2009). Clock genes have been identified in *Ae. albopictus*, and transcriptional profiling shows higher levels of *tim* and *cry1* transcripts in whole bodies of non-blood-fed females reared under short day conditions compared to those reared in long day conditions (Huang et al., 2015). This suggests that diapause-inducing photoperiod modulates the expression of *tim* and *cry1* genes. Although diapause-inducing short day conditions alter maternal clock components, their roles in diapause induction may be independent from their role in the circadian function, as has been proposed for *tim* (Bradshaw and Holzapfel, 2010). Although molecular clock components and central brain anatomy are generally conserved between diapausing and non-diapausing insects (Figures 2B,C) and recent work has mapped the clock neuron anatomy in *Ae. aegypti* and *An. coluzzi*, the circadian clock circuitry and the molecular mechanisms underlying the translation of seasonal cues to diapause responses in *Ae. albopictus* remain uncharacterized (Summa et al., 2012; Baik et al., 2020). Whether allelic differences in clock genes are present in different *Ae. albopictus* populations and whether these associate with diapause behaviors (as in *D. triauraria*) also remains unstudied.

Clock genes may affect downstream metabolic pathways to appropriately allocate energy reserves. This suggests that short photoperiod is detected by clock cells in the brain of *Ae. albopictus* females and this signal is translated into hormonal cues that determine the diapause fate of her offspring. A connection between the circadian system and insulin signaling pathways is observed in *D. melanogaster*, where PDF and short Neuropeptide F (sNPF) inhibit reproductive dormancy by modulating insulin producing cells. Furthermore, genetic manipulations of PDF-expressing neurons, including the sNPF-producing small ventral Lateral Neurons (s-LNvs), affect reproductive arrest (Nagy et al., 2019). This suggests that neural connections between the clock and reproductive systems may be critical for coordinating egg provisioning and development, but whether these connections exist in *Ae. albopictus* and whether their modulation is dependent on diapause state are areas for future research.

## Pathways Involved in Diapause Egg Production: Diapause Preparation and Initiation

Diapause induction involves the alteration of signaling processes in adult females prior to the developmental arrest of their offspring. Recent studies performing RNA-seq high-throughput sequencing have generated a global transcriptome analysis of blood-fed and non-blood-fed adult female *Ae. albopictus* reared in diapause and non-diapause inducing conditions (Huang et al., 2015). The transcriptional profiles of these mosquitoes undergo drastic changes in pathways involved in blood digestion, hormone synthesis, vitellogenin synthesis, insecticide resistance, and the circadian clock system. In non-blood-fed females reared in diapause-inducing conditions, potential regulatory elements of diapause induction (i.e., transcripts in pathways related to energy production and nutrient provisioning) were upregulated compared to both non-blood-fed females reared under long day conditions (Huang et al., 2015). These findings indicate that, in response to environmental conditions that induce diapause, females undergo changes in their nutrient provisioning pathways to appropriately produce diapause eggs.

### Cell Cycle Arrest

During diapause, development is arrested at a fixed stage. Specifically, cells in target organs, including the primordial imaginal structures in larval and pupal stages, halt their differentiation and progression through the cell division cycle (Nakagaki et al., 1991; Tammariello and Denlinger, 1998). Cell cycle arrest is one of the unifying themes of diapause, and positive cell cycle regulators and DNA replication-associated transcripts are downregulated during diapause induction in *Ae. albopictus*. Notably, transcripts for the positive cell cycle regulator, *proliferating cell nuclear antigen (pcna)*, are downregulated and similar patterns of *pcna* expression are reported during diapause induction in flesh flies, drosophilids, cotton bollworms, and apple maggots (Flannagan et al., 1998; Koštál et al., 2009; Bao and Xu, 2011; Ragland et al., 2011). The exact phase of cell cycle arrest varies between species. The flesh fly *Sarcophaga crassipalpis* undergoes larval diapause with most brain cells in G0/G1 phase (Tammariello and Denlinger, 1998), whereas diapausing *Bombyx mori* embryos have cells that are halted in G2 phase of cell division (Nakagaki et al., 1991). In the jewel wasp *Nasonia vitripennis*, 80% of cells halt at G0/G1 and 20% halt in G2 phases, a notably high proportion of subdominant cell cycle phase (Shimizu et al., 2018). Similarly, transcripts associated with cell proliferation are down-regulated in female *Ae. albopictus* under diapause-inducing conditions even prior to blood feeding, presumably to allocate energy for alternative metabolic pathways (Huang et al., 2015). These findings indicate that critical cellular processes are modulated in the adult female well in advance of the actual developmental arrest of her offspring.

### Insulin/FOXO Signaling

The insulin signaling/FOXO (*forkhead transcription factor*) pathway has been connected with many features of diapause including: arrested reproduction, extended lifespan, metabolic suppression, fat hypertrophy, and enhanced stress tolerance (Sim and Denlinger, 2013). The insulin pathway is employed in other types of reproductive arrest including dauer formation in *Caenorhabditis elegans* (Gottlieb and Ruvkun, 1994). PI3K is a component of the insulin-signaling pathway with naturally occurring variants that segregate with the ability to enter adult diapause in *D. melanogaster* (Williams et al., 2006). Interestingly, transcriptome analysis of *Ae. albopictus* oocytes showed that targets of FOXO are upregulated in females reared in diapause-inducing conditions, suggesting an important role for FOXO during the early stages of diapause egg production (Poelchau et al., 2011). Further evidence in *Culex* mosquitoes indicates that insulin signaling acts through downstream FOXO pathways to appropriately stockpile lipid reserves (Sim and Denlinger, 2008). Cold and desiccation tolerance are also important components for the survival of diapause eggs that may be linked to insulin signaling. Cold tolerance is achieved through upregulation of protective antioxidant enzymes that are regulated by FOXO in mosquitoes (Sim and Denlinger, 2011). Corazonin and CAPA neuropeptides affect the resistance to metabolic and desiccation stress in *D. melanogaster* and may also play roles in mediating desiccation resistance in diapause eggs (Zandawala et al., 2021).

Although multiple insulin-like peptides (ILPs) are encoded in *Aedes* and *Culex* genomes, ILP1 (but not ILP5) has been implicated in mediating diapause in *Culex* through the insulin receptor, suggesting that ILPs play distinct roles. Together, these studies suggest a model in which short days lead to a shutdown in insulin signaling that subsequently releases the repression of *FOXO*, leading to diapause induction. To fully understand the influence of insulin pathways in diapausing insects, it will be necessary to characterize the spatiotemporal expression and release of ILPs as well as their distinct or combinatorial regulation of lipid storage and cold tolerance (Sim and Denlinger, 2009). The *Ae. albopictus* genome encodes homologs of both ILP1 and ILP5 (Clark et al., 2016), although their roles in diapause entry remain untested. The involvement of ILP pathways in growth and development suggests that future studies to understand the specific role(s) of insulin signaling in diapause will require targeted spatial or temporal manipulations to limit these disruptions to specific timepoints and/or tissues of interest.

### Nutrient Sensing and Storage Pathways

**FIGURE 3:**
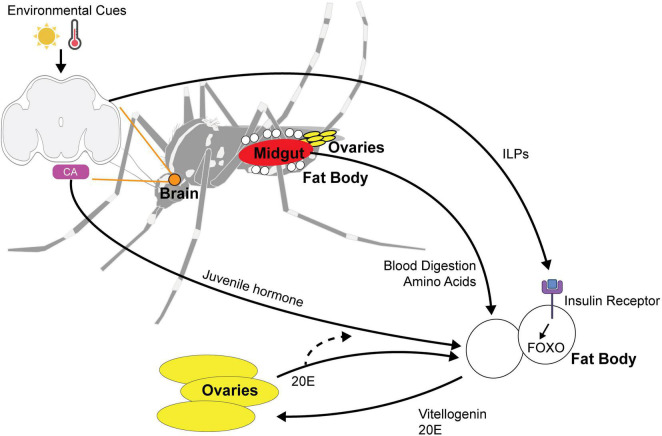
Multi-organ signaling pathways for maternally instigated diapause in female *Ae. Albopictus*. Environmental cues such as photoperiod and temperature are sensed by the central brain and signal the fat body using (1) ILPs (insulin-like peptides) and (2) juvenile hormone released by the corpora allata (CA). ILPs bind to insulin receptor and activate FOXO pathway. Blood nutrients are digested by the midgut and the integration of environmental cue and blood-fed status trigger the release of vitellogenin and 20E signaling to the ovaries to alter reproduction in diapause state. 20E feedback to the fat body and is likely to affect juvenile hormone biosynthesis.

*Aedes albopictus* females generally require protein from a blood meal to develop their eggs, although autogenous strains have been reported to produce eggs without a blood meal (Hawley, 1988; Chambers and Klowden, 1994). *Ae. albopictus* diapause eggs are larger and contain approximately 30% more lipids than non-diapause eggs, presumably due to an upregulation of lipid storage genes and a downregulation of lipid mobilization genes during the diapause initiation period (Reynolds et al., 2012). The identity of lipids also differs, with diacylglycerides and triacylglycerides being particularly abundant in diapause eggs (Batz and Armbruster, 2018). This suggests that blood nutrients must be utilized differently by the female to produce diapause eggs, which are more energetically costly. Nutrient utilization involves coordination between multiple tissues including the gut, ovaries, and fat body (Figure 3). The fat body is an organ that plays similar roles to the mammalian liver and is crucial for nutrient sensing, lipid storage, and endocrine signaling to the brain and reproductive organs.

Prior to blood feeding, ovary maturation remains in a previtellogenic state of arrest and the production of vitellogenin, the major yolk protein precursor, is repressed by the fat body. After a blood meal, multiple factors trigger vitellogenin protein synthesis in the fat body. Vitellogenins are then released into the hemolymph and taken up by the developing oocytes (Hansen et al., 2004, 2014; Attardo et al., 2005). Even before blood feeding, diapause-inducing conditions increase the expression of genes involved in amino acid synthesis and metabolism, suggesting that short day exposure primes females to differentially utilize blood protein compared to long day exposure (Huang et al., 2015). Interestingly, alanine-specific transferases are upregulated in females exposed to short day conditions that have not yet blood fed. In *B. mori*, alanine has been proposed to play a unique role in cryo-protection of diapause eggs, potentially revealing a requirement for females to provision their eggs with this specific amino acid (Suzuki et al., 1984). Furthermore, blood-fed females raised in diapause-inducing conditions have increased degradation of valine, leucine, and isoleucine, suggesting that amino acid handling may undergo changes during preparation to lay diapause eggs. Females reared in diapause-inducing conditions also demonstrate an upregulation in global metabolic pathways (phosphoenolpyruvate carboxykinase transcript), fatty acid metabolism (fatty acid synthase, fatty acid desaturase, delta(9)-desaturase 2), transcripts related to blood digestion (*trypsin*), detoxification (*glutathione transferase*, *thioredoxin peroxidase*), stress response, 20-hydroxyecdysone (20E) synthesis (*CYP302A1* and homolog of *Spook*), and vitellogenesis (Vitellogenin-A1 precursor) in response to a blood meal. Notably, upregulation of the vitellogenin synthesis gene *PVG1* was greater in females living in diapause-inducing conditions (Huang et al., 2015). This suggests that, although the general digestive and reproductive functions associated with blood feeding are similar, females show alterations in gene expression levels that allow them to differentially utilize nutrients for the production of diapause eggs. Although *Ae. albopictus* females reared under short day conditions have larger body sizes, no association between egg production per female, lifespan, or blood feeding propensity has been found (Costanzo et al., 2015). However, diapause status of females was not tested in this work, and systematic study of host-seeking or blood-feeding behavior to determine whether biting frequency or meal size(s) differ in females preparing to lay diapause vs. non-diapause eggs remains uncharacterized.

### Juvenile Hormone Signaling

Hormonal control of diapause is a common theme in insects and, in preparation for laying diapause eggs, females must coordinate JH, 20E, and insulin signaling to appropriately provision their offspring. Juvenile hormone (JH) plays critical roles in development and reproduction and has been linked to adult diapause in insects (Spielman, 1974; Saunders et al., 1990). Previous work in *Culex* mosquitoes demonstrated that long nights (short daylength) suppress JH synthesis through intermediate suppression of insulin signaling (Sim and Denlinger, 2013). In *D. melanogaster*, neuropeptides produced by circadian clock cells (PDF and sNPF) signal to insulin-producing cells (Nagy et al., 2019). In *D. melanogaster*, insulin receptors are present on the corpora allata (the tissue that synthesizes JH) and disruption of the insulin receptor also alters key enzymes required for JH synthesis (Belgacem and Martin, 2007).

Before a blood meal, JH induces the primary follicles to enter a resting stage, priming the fat body for vitellogenin synthesis as soon as a blood meal is consumed (Clements, 1999). In non-blood-fed females, genes encoding putative JH-inducible proteins are upregulated under short day conditions (Huang et al., 2015) and elevated JH-induced signaling likely enhances the fat body’s competence for vitellogenin synthesis, potentially increasing vitellogenesis for additional nutrient provisioning of diapause eggs once a blood meal is consumed. The overall transcriptional responses to a blood meal are similar under both short and long day conditions including the upregulation of PVG1 and trypsin genes, which reflects the transcriptional upregulation of vitellogenesis, blood digestion, and detoxification genes (e.g., glutathione S-transferase and thioredoxin peroxidases), consistent with previous studies (Ribeiro, 2003; Sanders et al., 2003; Dana et al., 2006).

Juvenile hormone synthesis may also be regulated by steroid hormone levels associated with egg development; recent work in cabbage beetles demonstrated that levels of 20-Hydroxyecdysone (20E), a steroid hormone critical for egg development, are regulated by environmental cues and that 20E affects JH biosynthesis and reproductive diapause in adult females (Guo et al., 2021). Cytochrome P450 monooxygenases are a superfamily of enzymes involved in hormone synthesis and insecticide resistance (Hlavica, 2011). 20E-synthesizing Cytochrome P450 enzyme (CYP302A1) and the homolog of Spook are upregulated in response to a blood meal under both long and short day conditions, consistent with the role of 20E in promoting egg development (Clements, 1999; Huang et al., 2015). However, CYP314A1, which encodes an enzyme that catalyzes the final step in conversion of ecdysone to 20E, is uniquely upregulated in blood-fed females under diapause-inducing short day conditions (Huang et al., 2015). Although the overall transcriptional responses are similar and suggest that nutrients are used to support vitellogenesis under both short and long days conditions, changes in maternal hormone signaling may tailor egg production for the increased nutritional demands of diapause eggs.

After a blood meal JH-inducible proteins are generally downregulated during non-diapause reproduction; however, blood-fed females under short-day conditions showed upregulation of four putative JH-inducible proteins (Shapiro et al., 1986; Huang et al., 2015). This upregulation of JH-induced signaling under diapause-inducing conditions in blood-fed females suggests that reproductive endocrine signaling is altered during diapause induction. Interestingly, diapause eggs themselves show reduced levels of JH (Batz et al., 2019).

These findings suggest potential mechanisms for the integration of environmental cues, sensed by the circadian system and reproductive status, reflected in 20E levels, to affect JH signaling. Further investigation to understand how these hormones regulate diapause will be challenging as they are critical for both embryonic development and molting and will require tools to manipulate JH pathways with spatial and temporal precision.

## Signatures of Diapause Eggs: Diapause Maintenance

Diapause eggs undergo drastic morphological, physiological, and metabolic changes to survive the winter. Diapause eggs have reduced metabolism for long-term survival and are larger in size, particularly in width and volume, due to increased egg lipid reserve (Wang, 1966; Lacour et al., 2014). Diapause eggs of *Ae. albopictus* also become desiccant- and cold-resistant and are capable of tolerating temperatures as low as –10°C (Hanson and Craig, 1995). This enhanced desiccation resistance is associated with increased surface hydrocarbons (Urbanski et al., 2010). Scanning and transmission electron microscopy analysis of egg composition showed that the dark endochorion layer shrinks, likely due to the compaction of fatty acids that creates a physical barrier against ice formation, and that the serosal cuticle, which secretes the waxy layer, thickens for stronger sclerotization and chitinization (Kreß et al., 2016). Egg hardiness is likely due to both qualitative and quantitative changes of the egg shell.

Transcriptome data of oocytes destined to become diapause or non-diapause eggs show drastic changes in functional pathways that regulate metabolism, cell maintenance, and endocrine signaling (Poelchau et al., 2013a). Diapause eggs have decreased metabolism, which is reflected by the abundance of phosphoenolpyruvate carboxykinase transcript (*pepck*) in diapause-destined oocytes. PEPCK enhances the gluconeogenesis pathway to switch over to anaerobic metabolism. During metabolic suppression, many animals decrease aerobic metabolism and shift largely to anaerobic metabolism, favoring the activity of glycolysis and gluconeogenesis, the pentose phosphate shunt, and the PEPCK-succinate pathway to generate ATP (Hahn and Denlinger, 2011). PEPCK overexpression could reflect a maternally provisioned regulatory cue or the initiation of the gluconeogenic pathway in preparation for diapause (Hahn and Denlinger, 2011; Poelchau et al., 2013a). The *pepck* transcript is upregulated in non-blood-fed females reared in diapause-inducing conditions, suggesting that it may also play a role in metabolism prior to egg production (Huang et al., 2015).

Diapause as a state of developmental arrest is reflected by cell cycle arrest and accompanying overexpression of transcripts involved in DNA replication and transcription. In particular, *inhibitor of growth protein* (*ing1*, AALF016435; LOC109399745) and *bhlhzip transcription factor bigmax* (AALF005213; LOC109405346) are abundant in diapause-destined oocytes. In *D. melanogaster*, ING1 likely interacts with the transcription factor *p53*, which regulates cell-cycle arrest in response to stress (Lunardi et al., 2010). The *Bhlhzip bigmax* transcription factor is involved in metabolism and energy sensing pathways and is a likely target of FOXO, an important regulator of diapause in *Cx. pipiens* that functions downstream of insulin signaling (Sans et al., 2006; Sim and Denlinger, 2008; Alic et al., 2011).

Lastly, transcriptomic profiles of endocrine signaling pathways showed increased levels of *rack1* activated protein kinase C receptor and decreased levels of ecdysone inducible protein L2 (*eip*) in diapause oocytes (Poelchau et al., 2011). RACK1 is reported to have a role in ecdysone signaling, which is critical for vitellogenesis, and is differentially expressed in diapausing cricket embryos (*Allonemobius socius*) (Quan et al., 2006; Reynolds and Hand, 2009). Moreover, RACK1 is an integral component of the mammalian circadian rhythm circuitry in mice (Robles et al., 2010), consistent with the hypothesis that photoperiod is the likely environmental cue initiating diapause transition. This suggests RACK1 may play roles in environmental cue detection by the female as well as diapause induction in her eggs. The *eip* homolog *imp-l2* in *D. melanogaster* is important for neural and ectoderm development (Garbe et al., 1993) and is essential for enduring periods of starvation (Honegger et al., 2008). Furthermore, both *rack1* and *eip* are implicated for regulating the size of ovaries in *D. melanogaster* (Kadrmas et al., 2007; Honegger et al., 2008) and may act similarly in regulating the size of diapause eggs in *Ae. albopictus*.

Hormonal signaling pathways are also likely contributing to diapause maintenance in *Ae. albopictus* embryos independently from their roles in adult females. Diapause eggs show lower levels of JH and JH pathway-associated transcripts compared to non-DP eggs when measured using LC-MS, which suggest that JH levels are reduced in diapause (Poelchau et al., 2013b; Batz et al., 2019).

Although diapause maintenance may appear to be an inactive period, early vs. late diapause embryos show distinct gene expression profiles—highlighting the dynamic nature of diapause (Poelchau et al., 2013b).

## Diapause Termination

Although our understanding of the mechanisms of diapause termination are incomplete, *Ae. albopictus* embryos terminate maternally instigated diapause after a period of time to enter the “quiescence” phase, in which they become responsive to cues that signal the return of favorable conditions and development resumes (Batz et al., 2020). If unfavorable conditions persist, development may remain suppressed in post-diapause quiescence but this form of dormancy is distinguishable from diapause because it can be terminated immediately upon exposure to favorable conditions (Tauber and Tauber, 1976). In temperate conditions, embryos often terminate diapause during the winter, thus regaining responsiveness to environmental cues while still suppressing their development in the quiescence phase. This can lead to a stockpile of eggs that are primed to hatch when favorable conditions return, resulting in a relatively synchronous springtime emergence (Lacour et al., 2015). Non-diapause embryos also enter quiescence if hatching stimuli are not present immediately after embryonic development; however, these eggs hatch as soon as they receive an appropriate stimulus (Judson et al., 1965).

Stimuli often used to terminate *Ae. albopictus* embryonic diapause in the laboratory include exposure to long daylengths, direct application of JH and chilling (not freezing) temperature (Spielman, 1974). In the field, *Ae. albopictus* eggs terminate diapause and enter quiescent phase in advance of the return of favorable conditions, usually indicated by the low temperatures and extended photoperiods associated with early spring (Vinogradova, 2007; Lacour et al., 2015). Consistent with the finding that JH abundance is reduced during diapause, the juvenile hormone-analog, pyriproxyfen, can also terminate diapause when directly applied (Suman et al., 2015; Batz et al., 2019). Although hatching is generally measured as the binary output of diapause exit, diapause is a dynamic state and both photoperiod and temperature can be thought of as regulating the *rate* of diapause development under natural conditions. As diapause progresses, gene expression patterns tend to become more similar to those of quiescent embryos, supporting the concept of diapause as a dynamic process and suggesting that diapause duration may be endogenously timed (Poelchau et al., 2013b). Although *Wy. smithii* uses daylength as a cue for termination (Bradshaw and Lounibos, 1977), few species have been shown to require a specific stimulus to end diapause. Laboratory studies often show that long daylengths can terminate both laboratory- and naturally induced autumnal diapause. However, it may be misleading to assume that long daylength serves as a direct termination signal considering that quiescence dormancy phase is maintained during longer daylength. This suggests diapause termination may occur after a predetermined phase, induced by a combination of environmental cues, or initiated by the integration of daylength dynamics, depending on the species.

Female *Ae. albopictus* that produce diapause eggs are unlikely to survive winter conditions and are presumed to die. Whether these adult *Ae. albopictus* females are capable of switching back to non-diapause egg production remains unexplored. The accuracy of behavioral changes as indicators of natural diapause termination is dependent on how closely timed are the behavioral output to the reactivation of the endocrine system (Tauber and Tauber, 1976). Interestingly, species in which adults enter and exit diapause themselves show altered behaviors and genetic expression patterns post-diapause. For example, in some tick and mosquitoes species that enter diapause as adults, the readiness to consume a blood meal is used as a behavioral indicator to determine the end of diapause (Wilkinson, 1968; Washino, 1970; Spielman and Wong, 1973). In *Cx. pipiens* females, which enter diapause as adults, *cry2* expression profile is changed after diapause termination and may serve as a biomarker for other diapausing insects (Meuti et al., 2015).

## Future Research Directions

While extensive work has been carried out to characterize the differences between diapause and non-diapause eggs and the environmental cues sufficient to induce this period of suspended development, there is a major gap in our understanding of how adult females translate seasonal cues to initiate diapause egg production. Specifically, the mechanism by which gradual environmental changes are sensed by the female and translated into a binary reproductive switch remains unknown.

The variation in diapause physiology and behavior of *Ae. albopictus* populations across global habitats provides a unique system to understand the adaptation of seemingly similar nervous system circuitry to different environmental conditions. Furthermore, *Ae. albopictus* serves as a unique biological model to study the maternal effects on the fate of her over-wintering progeny to maximize survival. It is possible that mothers reprogram the developmental timeline of their offspring through epigenetic mechanisms but this remains unstudied.

New discoveries and tools developed in *Ae. albopictus* could be applied to study other invasive species that undergo distinct forms of reproductive diapause arrest. By expanding the field’s toolkit, these strategies could be applied to study and control a broad range of disease vectors and crop pest species.

### Genome Editing

Whole genome sequencing of *Ae. albopictus* (Palatini et al., 2020) and the emergence of new genome editing tools allow for the unprecedented ability to manipulate genes and cells to examine their role in regulating diapause (Riabinina et al., 2015; Chaverra-Rodriguez et al., 2018). Piggybac- and PhiC31 genetic transformation has been demonstrated in a number of mosquito species, including *Ae. albopictus* (Labbé et al., 2010) and CRISPR-Cas-based genome editing has reliably generated knock-in and knock-out mutants in multiple mosquito species including: *Ae. albopictus, Ae. aegypti*, *An. gambiae*, and *Cx. quinquefasciatus* (Kistler et al., 2015; Anderson et al., 2019; Liu et al., 2019; Macias et al., 2019). Additionally the Q binary system has been successfully applied in *Ae. aegypti* and *An. gambiae* (Riabinina et al., 2016; Matthews et al., 2019), which allows for the application of a genetic toolkit to label and manipulate defined subsets of cells and is now poised to be applied in other species.

Recent work has identified chromosomal regions enriched for SNPs that differ between temperate and tropical strains (Boyle et al., 2021) and candidate genes in these regions could provide a basis for investigating the genetic regulation of diapause using targeted genome-editing. However, previous work suggests that generating disruptive mutations to a single locus will likely be uninformative to identify the genes regulating diapause in *Ae. albopictus*. Evolutionary history suggests diapause is polygenic and would likely require multiple genetic manipulations to alter diapause behavior (Boyle et al., 2021). Additionally, most candidate genes for controlling diapause entry are involved in many essential physiological processes (i.e., insulin signaling) and globally disrupting these genes is expected to cause lethality and pleiotropic effects. Genetic tools that allow spatial (i.e., tissue-specific drivers) or temporal (i.e., inducible drivers) control over transgene expression will likely be required to overcome pleiotropy associated with many of these genes to understand the specific roles that they play in diapause. “Split” binary systems, including the Q system, represent one approach to limit the expression of effectors to a restricted subset of cells by “splitting” the QF2 transcription factor into two parts with the DNA binding domain and activation domain expressed under the control of different drivers. This means that functional QF2 is only produced in cells that co-express both domains (Riabinina et al., 2019; Younger et al., 2022). These tools could be applied for targeted manipulation of developmentally essential genes only in restricted subsets of cells. For example manipulating only insulin receptor-expressing cells within the clock system by combining *InR* and *per* split driver lines. However, this approach relies on the identification and validation of driver lines to allow genetic subpopulation sectioning.

Temporal control of experimental manipulations may be even more critical for understanding the roles of specific signaling pathways or cells in diapause. Optogenetic and chemogenetic tools allow researchers to activate or silence cells of interest with temporal control depending on when the exogenous stimulus is applied. These have recently been deployed in *Ae. aegypti* (Jové et al., 2020; Sorrells et al., 2021), but may not be ideal for the long timescale of activation associated with responses to environmental cues that change over the course of days. The development and application of experimentally inducible transgenes in mosquitoes also provides potential avenues for understanding the roles of fundamental genes in specific behaviors by disrupting or rescuing gene function with temporal specificity (Chen et al., 2021). For example, these tools could allow researchers to manipulate maternal insulin-producing cells specifically during diapause induction and egg provisioning, while leaving the insulin signaling intact during development.

While forward genetic screening is currently too low-throughput and labor intensive to be feasible, candidate and RNAi-base approaches have been successfully applied in *Ae. albopictus* and could allow for temporally specific disruption of targets of interest (Xu et al., 2018).

### Mapping and Characterizing Maternal Diapause Induction Circuitry

As noted above, the ability for female *Ae. albopictus* to optimize their behavior and reproduction by integrating internal metabolic and reproductive states with external environmental conditions is crucial for species propagation. The exact circuitry and mechanisms underlying this adaptive process remain unclear. Genetic and technological advances in the field now allow researchers to identify cells that respond to environmental cues to understand the mechanisms underlying diapause development, physiology, and behavior. A possible circuit may consist of detection of environmental cues by either sensory neurons that relay that information to clock cells or circadian clock cells themselves, which in turn release neuropeptides to control circulating levels of ILPs and signal to the distant tissues, including the fat body and ovaries, to alter nutrient utilization and JH levels. Mapping the anatomical and functional connections between cells that detect seasonal cues and those that control nutrient allocation is a critical first step to connect the female’s response to environmental cues with her reproductive physiology.

Using reporters such as GCaMP, researchers can measure the acute activity of target cells in response to environmental cues during the critical period of diapause transition. The functional role of target cells may be directly tested using genetic or pharmacological manipulations to determine if diapause can be effectively induced or if these manipulations are sufficient to block environmentally induced diapause. The application of these cutting-edge genetic tools in *Ae. aegypti* has established their feasibility and the field is now poised to anatomically and temporally map diapause-inducing circuitry in *Ae. albopictus* and to compare these neural circuits in different behavioral states (diapause vs. non-diapause egg production) and between populations with varying diapause competence (Jové et al., 2020; Zhao et al., 2021).

### Metabolic Profiling of Diapause Preparation

Although metabolomic methods have been applied to study insecticide resistance and embryonic diapause, the changes in metabolic profiles of female *Ae. albopictus* preparing to lay diapause eggs has not yet been characterized (Huang et al., 2015; Batz and Armbruster, 2018). Untargeted metabolomic technology allows researchers to simultaneously assess amino acids, lipids, polyols, fatty acids, and metabolic intermediates in insect and have demonstrated that diapause eggs show distinct metabolic profiles compared to non-diapause eggs with particular enrichment of diacylglycerides and triacylglycerides (Colinet et al., 2012; Batz and Armbruster, 2018). Untargeted metabolomic approaches provide an unbiased snapshot of organismal physiology and may identify unexpected metabolic changes that can be used as the basis for subsequent targeted experiments, although they lack the specificity and pathway coverage of targeted analysis (Macel et al., 2010). Additionally, the metabolites that are detected vary depending on which extraction and separation methods are used (Cajka and Fiehn, 2016). Targeted metabolic profiling is available to measure triglyceride/lipoprotein levels in adult females from laboratory and field populations preparing to lay diapause and non-diapause eggs using assays such as the colorimetric sulfophosphovanillin (SPV) (Knight et al., 1972; Van Handel, 1985; Men et al., 2016) or the Glycerol-3-phosphate Oxidase (GPO) with *N*-ethyl-*N*-(2-hydroxy-3-sulfopropyl)-3,5-dimethoxyaniline sodium salt (DAOS) methods (Sawabe and Moribayashi, 2000). Both are attractive methods to quantify the total lipid reserves from single adult females as they are fast- and high-throughput assays and have been used to measure total lipid content in other mosquito species and *D. melanogaster.* Metabolic changes correlated with diapause behaviors will yield new insight into how the brain integrates metabolic and reproductive status to undergo reproductive switch at appropriate seasons.

### Maternal Behaviors in Preparation for Diapause Egg Production

Many outstanding questions remain regarding how adult females detect seasonal cues to initiate the drastic switch to diapause state. As obligate blood-feeders, females consume a blood meal to obtain the protein required to complete reproduction and lay eggs. The effects of diapause-inducing conditions on the host-seeking and blood-feeding behaviors of adult females are poorly characterized. Using validated assays, the differences in host-seeking, blood feeding, and engorgement behaviors can be compared between females preparing to lay diapause and non-diapause eggs. Previous studies suggest while *Ae. albopictus* females suppress their drive to find and bite hosts in the days following a blood meal, they are capable of multiple host-feedings without laying eggs, particularly if they have a smaller body size (Klowden and Briegel, 1994; Farjana and Tuno, 2013). Females preparing to lay diapause eggs may require multiple bouts of blood feeding to obtain the nutrient levels necessary to lay lipid-rich diapause eggs and may also show altered host-seeking regulation.

A recent study characterized egg laying behavior of *Ae. albopictus* reared in favorable and unfavorable conditions (Reinbold-Wasson and Reiskind, 2021). They focused on “skip-oviposition” behavior, broadly defined as female mosquitoes distributing eggs among multiple oviposition sites during a single gonotrophic cycle. In both favorable and unfavorable conditions, *Ae. albopictus* females spread their eggs widely, suggesting that this egg-laying behavior is unaltered by diapause state. However, other groups have reported that females distribute their eggs more broadly across oviposition sites when tested in summer conditions compared to fall conditions (Fonseca et al., 2015). Further critical assessment of feeding and reproductive behaviors in females reared in diapause-inducing conditions could identify new potential targets to disrupt these behaviors or more efficient ways to deploy mosquito control strategies throughout the year, for example if oviposition sites are reliably denser in the fall compared to the summer.

### Ecology and Population Biology

The differences in reproductive physiology and behavior of females in temperate and tropical conditions are interesting examples of evolutionary divergence. These temperate and tropical populations share similar genetic components, neural circuitry, and anatomy yet produce distinctly different behavioral outputs in response to environmental conditions. These differences raise intriguing research questions about the genetic and neural basis of maternally induced diapause and the plasticity of these behaviors.

As climate change and urbanization open new areas for territorial expansion, tracking and characterizing new invasions around the globe may provide opportunities to examine the plasticity of diapause and to uncover the mechanisms by which these selective pressures shape behavior. These studies could address the conflicting observations on whether diapausing ability is gained or lost throughout history. Furthermore, it could yield insights into the evolutionary timeline of the emergence and disappearance of adaptive behaviors induced by environmental changes, especially as climate change contributes to the expansion of this species’ geographical range (Ryan et al., 2019). Work in *Wy. smithii* has demonstrated genetically based shifts in CPP toward shorter more “southern” CPPs in populations in more northern latitudes – consistent with an adaptive response to prolonged warm season (Bradshaw and Holzapfel, 2001). Altogether, these findings suggest that *Ae. albopictus* populations that are able to adapt to harsh winters are poised to expand their geographic ranges. Furthermore, *Ae. albopictus* diapause affects seasonal species abundance and this can influence inter-species interactions and competition.

### Interaction With Artificial Environmental Conditions

Due to global urbanization, the increased presence of artificial light at night (ALAN) has greatly impacted the diapause physiology and behavior of temperate mosquito strains. A recent field study showed that ALAN exposure interferes with daylight as a cue for seasonal dynamics and significantly reduced diapause incidence in *Ae. albopictus* (Westby and Medley, 2020). Similarly, diapausing adult female *Cx. pipiens*, which undergo reproductive arrest, inappropriately averted diapause state by becoming reproductively active when exposed to ALAN in laboratory settings (Fyie et al., 2021). In these females, whole body fat content was significantly reduced, egg follicles were larger, and blood-feeding increased (Fyie et al., 2021). Likewise, *Mamestra brassicae* caterpillars exposed to low intensities of artificial light at night as larvae showed disrupted pupal diapause initiation as moths (Van Geffen et al., 2014). ALAN exposure likely interferes with photoperiod dynamics as an environmental cue for seasonal timing, resulting in improperly initiated diapause. As *Ae. albopictus* continues to expand its range, urbanization and ALAN will be important factors that determine seasonal biting patterns and the risk of disease transmission. From a basic science perspective it will be interesting to understand how these factors interact with the neural circuitry that controls diapause.

## Applications for Mosquito Control and Public Health

*Aedes albopictus* poses increasing threats to public health largely due to its ability to live in a broad geographic range and to vector a number of arboviruses that threaten human health (Ibáñez-Bernal et al., 1997; Paupy et al., 2012; Lindh et al., 2019). Diapause can lead to the synchronized seasonal abundance of *Ae. albopictus*, which can contribute to interspecific competition and could directly influence pathogen transmission cycles (Joy and Sullivan, 2005). In temperate regions, predictable seasonal changes in the incidence of larval vs. adult mosquitoes allow vector control strategies to be efficiently targeted to specific life stages at certain times of year. For example, prioritizing larvicidal control in the early- to mid-spring will be particularly effective as eggs exit diapause, enter quiescence and hatch because there are relatively few adult mosquitoes present at this time of year (Figure 1B; Lacour et al., 2015).

Understanding diapause pathways in *Ae. albopictus* could facilitate the development of new methods of mosquito control by creating tools to disrupt reproductive and/or vectorial capacity. For example, deploying compounds that block diapause entry or promote inappropriate diapause exit could reduce mosquito populations in temperate conditions. Alternately, finding ways to inappropriately induce diapause in tropical conditions could also suppress the populations.

New control strategies are urgently needed as this species continues to expand its range (Figure 1A). The risk of severe outbreaks of emerging vector-borne diseases is an increasing threat for more of the world (Bonizzoni et al., 2013; Ryan et al., 2019). Targeting the pathways that control and optimize reproduction may be a particularly effective approach to reduce mosquito populations, prevent vector-host interactions, and limit the spread of vector-borne disease.

## Author Contributions

Both authors made direct and intellectual contribution to this work, wrote the manuscript, and approved it for publication.

## Conflict of Interest

The authors declare that the research was conducted in the absence of any commercial or financial relationships that could be construed as a potential conflict of interest.

## Publisher’s Note

All claims expressed in this article are solely those of the authors and do not necessarily represent those of their affiliated organizations, or those of the publisher, the editors and the reviewers. Any product that may be evaluated in this article, or claim that may be made by its manufacturer, is not guaranteed or endorsed by the publisher.
